# The response of canine faecal microbiota to increased dietary protein is influenced by body condition

**DOI:** 10.1186/s12917-017-1276-0

**Published:** 2017-12-04

**Authors:** Jia Xu, Adronie Verbrugghe, Marta Lourenço, An Cools, Daisy J. X. Liu, Tom Van de Wiele, Massimo Marzorati, Venessa Eeckhaut, Filip Van Immerseel, Lynn Vanhaecke, Miguel Campos, Myriam Hesta

**Affiliations:** 1 Department of Nutrition, Genetics and Ethology, Faculty of Veterinary Medicine, Ghent University, Heidestraat 19, 9820 Merelbeke, Belgium; 2 Present Address: Department of Agriculture and Bioengineering, Jinhua Polytechnic, Wuzhou steet 1188, 321007 Jinhua, Zhejiang Province People’s Republic of China; 3 Present Address: Department of Clinical Studies, Ontario Veterinary College, University of Guelph, 50 Stone Road E, Guelph, ON N1G 2W1 Canada; 4 Laboratory of Microbial Ecology and Technology (LabMET), Faculty of Bioscience Engineering, Ghent University, Coupure Links 653, 9000 Ghent, Belgium; 5 Department of Pathology, Bacteriology and Avian Diseases, Faculty of Veterinary Medicine, Ghent University, Salisburylaan 133, 9820 Merelbeke, Belgium; 6 Department of Veterinary Public Health and Food Safety, Faculty of Veterinary Medicine, Ghent University, Salisburylaan 133, 9820 Merelbeke, Belgium; 7 Department of Medicine and Clinical Biology of Small Animals, Faculty of Veterinary Medicine, Ghent University, Salisburylaan 133, 9820 Merelbeke, Belgium; 8 Department of Clinical Veterinary Science, Vetsuisse Faculty, Bern University, Länggassstrasse 128, 3001 Bern, Switzerland

**Keywords:** Butyrate-producing bacteria, Butyrate kinase, Canine, Obesity, Protein fermentation

## Abstract

**Background:**

High protein diets shift the faecal microbiota into a more unfavourable composition in obese humans. In lean dogs, higher protein consumption is accompanied with increased production of putrefactive fermentation products, whereas obese dogs have a different gut microbiota compared to lean dogs. Still, the impact of high dietary protein on gut microbiota in obese dogs remains unclear. The aim of this study was to investigate faecal microbial changes in lean and obese dogs in response to two different levels of dietary protein.

Six healthy lean and six obese Beagles were fed a high protein diet (HP) and a low protein diet (LP) for 28 days each in a crossover design. Denaturing gradient gel electrophoresis and quantitative PCR were performed on faecal samples for microbial profiling. Plasma acylcarnitine and fermentation metabolites were measured.

**Results:**

Dogs fed HP had higher concentrations of protein fermentation metabolites including faecal ammonia, isovalerate, isobutyrate, phenol, indole, serum indoxyl sulphate and plasma 3-OH isovalerylcarnitine compared to dogs fed LP, whereas no changes in faecal concentrations of acetate and butyrate were observed. The abundances of clostridial clusters IV and XIVa, covering the majority of butyrate-producing bacteria, and of the butyrate kinase gene, one of the terminal genes of the butyrate synthesis pathway were higher in dogs on HP compared to LP. Significant interactions between diet and body condition were found for the abundance of Firmicutes, *Lactobacillus* and clostridial cluster I. The similarity coefficient of faecal microbiota between the two diets was smaller in obese dogs than in lean dogs.

**Conclusions:**

High protein diet increased the abundance and activity of butyrate-producing bacteria in Beagles independent of the body condition. In addition, increasing dietary protein content had a greater overall impact on faecal microbiota in obese compared to lean dogs.

**Electronic supplementary material:**

The online version of this article (10.1186/s12917-017-1276-0) contains supplementary material, which is available to authorized users.

## Background

Obesity is defined as an accumulation of excessive amounts of adipose tissue in the body, and it is the most common nutritional disorder in companion animals [1]. The prevalence of obesity has been reported to range from 8 to 34% in dogs [2, 3]. In the last decade, the link between obesity and gut microbiota has been established in humans and rodents [4–6], however, compared to humans, the relationship between gut microbiota and obesity is less well understood in dogs and needs to be investigated [7, 8].

Gut microbiota are mainly influenced by undigested dietary carbohydrates and protein. In dogs, there is increasing interest in feeding high protein diets, e.g. raw meat-based diets and weight management diets [9, 10]. In obese humans, high protein diets result in decreased butyrate concentration and numbers of butyrate-producing bacteria. Additionally, several studies carried out in lean dogs reported negative effects of colonic protein fermentation including increased faecal pH and elevated production of putrefactive substances [11]. Furthermore, a high protein diet has shown to promote the growth of *Clostridium perfringens* and to reduce the abundance of clostridial cluster XIVa in dogs [12]. However, the impact of high protein diets on faecal microbiota in obese dogs has not been studied.

The interactions between gut microbiota and host metabolism are of great importance thus receiving increased attention more recently [13]. Short-chain fatty acids (SCFA) are major fermentation products that are rapidly absorbed and utilized by the host. The metabolism of SCFA requires activation with coenzyme (CoA) [14]. Intracellular CoA bound acylgroups are then transported from the cytoplasm to the mitochondria by means of carnitine groups. Therefore, acetylcarnitine, propionylcarnitine, and butyrylcarnitines are measures of the respective SCFA-CoA by which SCFA influence cellular metabolism [14, 15]. In addition, the protein fermentation product indole can be metabolized to indoxyl sulphate by the liver [16], and the latter has been associated with chronic kidney disease in dogs [17]. Therefore, the assessment of these metabolites might be a useful approach to evaluate host metabolism of the gut fermentation end-products [18, 19].

Butyrate-producing bacteria supply energy to the gut epithelium, regulate host cell responses, and therefore, are considered to exert health-promoting effects on the colon [20]. The reduction of butyrate-producing bacteria has been associated with colon cancer and inflammatory bowel disease [21, 22]. The biosynthesis of butyrate can occur via the butyrate kinase (BK) pathway or via the butyryl CoA: acetate CoA transferase (BCoAT) pathway [23]. Although clostridial clusters IV and XIVa consist the majority of butyrate-producing bacteria from human colon [24], they still habour a diverse collection of non-butyrate producers. Therefore, assessing terminal genes of butyrate synthesis pathways could provide valuable information specifically target the activity of the butyrate-producing bacterial community.

The aim of the present study was (1) to investigate the impact of dietary protein on faecal microbial profile and functionality especially focusing on butyrate-producing microbiota and the concomitant fermentation and host metabolic profile in dogs; and (2) to compare the response of lean and obese dogs to two different levels of dietary protein. We hypothesize that high protein diet alters the profile and functionality of faecal microbiota and thus induces associated changes to host metabolism. The diversity and composition of faecal microbiota were measured through DGGE and quantitative PCR. The functionality of faecal microbiota was assessed by quantifying faecal fermentation metabolites and terminal genes expression for butyrate synthesis. The effect on the host metabolism was evaluated through the concentrations of plasma acylcarnitines and serum indoxyl sulphate.

## Methods

### Animals and diets

This study was approved by the Ethical Committee of the Faculty of Veterinary Medicine, Ghent University, Belgium (EC 2011/056).

Twelve healthy Beagles (Marshall Farms and Domaine des Souches) with a mean age of 6.0 years (range 3.8–8.3 year) were included in this study. Six Beagles (one spayed and three intact females; two intact males) were lean with a body condition score (BCS) of 4–5/9 and six Beagles (three intact females, one castrated and two intact males) were obese with a BCS of 8–9/9 [25]. Obesity was induced approximately one year prior to the present study by feeding the dogs a high fat commercial diet as described by Van de Velde et al., [26]. Prior to the study, dogs were deemed healthy, apart from obesity in six dogs, based on physical exams, complete blood counts, and serum biochemistry.

Two isocaloric experimental diets (Table 1), a high protein diet (HP) which consisted of 50.0 g crude protein (CP), 12.2 g ether extract (EE) and 32.2 g nitrogen free extract (NFE) on 100 g dry matter (DM) basis and a low protein diet (LP) which consisted of 17.8 g CP, 13.6 g EE and 62.3 g NFE on 100 g DM basis were formulated with the same ingredients (NV Versele-Laga). Both diets met the Minimal Requirement for adult dogs according to the National Research Council (NRC) [27]. The initial amount of food offered was calculated based on individual maintenance energy requirements according to population history and adjusted to maintain a stable body weight throughout the study. Dogs were fed twice daily and had free access to water.

**Table 1:** Primer set used in the present study

Target	Primers (5’➔3′)	References
Bacteria V3 region	PRBA338f ACTCCTACGGGAGGCAGCAG	[56]
	PRUN518r ATTACCGCGGCTGCTGG	
Total Bacteria	fwd CGGYCCAGACTCCTACGGG	[57]
	rev TTACCGCGGCTGCTGGCA	
Firmicutes	fwd GGAGYATGTGGTTTAATTCGAAGCA	[58]
	rev AGCTGACGACAACCATGCAC	
*Enterobacteriaceae*	fwd CATTGACGTTACCCGCAGAAGAAGC	[59]
	rev CTCTACGAGACTCAAGCTTGC	
Bacteriodetes	fwd GGARCATGTGGTTTAATTCGATGAT	[58]
	rev AGCTGACGACAACCATGCAG	
*Lactobacillus*	fwd GGAATCTTCCACAATGGACG	[60]
	rev CGCTTTACGCCCAATAAATCCGG	
Clostridial cluster I	fwd TACCHRAGGAGGAAGCCAC	[61]
	rev GTTCTTCCTAATCTCTACGCAT	
Clostridial cluster IV	fwd ATGCAAGTCGAGCGA(G/T)G	[62]
	rev TATGCGGTATTAATCT(C/T)CCTTT	
Clostridial cluster XIVa	fwd CGGTACCTGACTAAGAAG	[63]
	rev AGTTT(C/T)ATTCTTGCGAAC	
Butyryl-CoA acetate-CoA transferase	fwd AAGGATCTCGGIRTICAYWSIGARATG)	[64]
	rev GAGGTCGTCICKRAAITYIGGRTGNGC	
Butyrate kinase	fwd TGCTGTWGTTGGWAGAGGYGGA;	[65]
	rev GCAACIGCYTTTTGATTTAATGCATGG	

### Animal experimental procedures

The study was designed as a crossover with two 4-weeks periods. The first 3 weeks were an adaptation period and samples were taken in the fourth week. In the first period, three lean and three obese dogs were randomly selected and assigned to LP first and the other three lean and obese dogs first received HP. In the second period, diets were switched. Each dog was therefore assigned to one of four groups (group 1: lean dogs received LP first; group 2 lean dogs received HP first; group 3 obese dogs received LP first; group 4 obese dogs received HP first).

### Sampling procedure

Leftover food was collected and weighed after each feeding. Body weight and BCS were measured weekly. After overnight fasting, blood samples were drawn from the jugular vein on day 27 of each period. Heparinized plasma and serum were obtained by centrifugation at 1620 *g* for 15 min at 4 °C and stored at −20 °C until assayed. On day 27 of each period, fresh faecal samples (± 10 g) were collected within 10 min after spontaneous voiding. An aliquot of ±2 g was put into a sterile plastic tube, frozen immediately on dry ice and stored at −80 °C for microbial analyses, and the rest was stored at −20 °C for chemical analyses.

### Chemical analyses

Dry matter of diets and faecal samples was analyzed by drying to a constant weight at 103 °C (ISO 1442, 1997), and crude ash was determined by combustion at 550 °C (ISO 936, 1998). Dietary crude protein was calculated from Kjeldahl nitrogen (6.25 × N, ISO 5983–1, 2005), ether extract was analyzed by the Soxhlet method (ISO 1443, 1973) and crude fibre was determined by acid-alkali digestion (ISO 5498, 1981). Nitrogen-free extract was calculated by subtracting crude ash, crude protein, crude fat, and crude fibre of the DM content. Total dietary fibre (TDF) and insoluble dietary fibre was measured with a Total Dietary Fibre Assay Kit (Sigma–Aldrich Co.), using procedures based on a combination of enzymatic and gravimetric methods [28]. Soluble dietary fibre was calculated by subtracting insoluble dietary fibre for the TDF [28].

Faecal pH was measured with a portable pH meter (Hanna Instruments). Faecal ammonia was analysed by steam distillation and titration [29]. Faecal SCFA concentrations were determined via gas chromatography after extraction with diethyl ether [30]. Faecal phenol, indole and p-cresol concentrations were measured as described by Depauw et al. [31].

Serum cobalamin and folate concentrations were measured using commercially available ARCHITECT B12 and ARCHITECT Folate assays, respectively, on ARCHITECT *i* System (Abbott Diagnostics). Plasma acylcarnitine profile was determined according to Zytkovicz et al. [32]. Serum indoxyl sulphate concentrations were measured according to Depauw et al. [31].

### Microbial analyses

Total bacterial DNA extractions from 500 mg faeces were performed according to Boon et al. [33]. Isolated DNA was subsequently used as a template to amplify the 16S rDNA for all members of the Bacteria with forward primer P338F-GC and the reverse primer P518r, and a GC-clamp of 40 bp was incorporated into the forward primer. DGGE based on the protocol of Muyer et al. [34] was performed on the Bio-Rad D gene system (Bio-Rad). The PCR products (10 μL of mixture from 20 μL PCR product and 5 μL loading dye) of the second round were loaded. The obtained DGGE patterns were normalized and analyzed using BioNumerics 2.0 (Applied Maths) [35]. The number of bands in the DGGE profile was used to calculate the richness in the present study. A matrix of similarities for the densiometric curves of the band patterns was calculated based on the Pearson product-moment correlation coefficient, and dendrograms were created by using Ward linkage [36].

The quantification of DNA by qPCR was performed with a C1000 Thermal Cycler (Bio-Rad). The amplification and detection were carried out in 96-well plates using SensiMixTM SYBR No-ROX Kit (Bioline Reagents Ltd). Each reaction was done in triplicate in 12 μL total reaction mixture using 2 μL of 50 ng of the DNA sample except for BK where 2 μL of undiluted DNA was used. All qPCR results were expressed as gene copies per g of fresh faeces. The primer sets used in this study are listed in Table 2. A melting curve analysis was done after amplification to confirm specificity of the reaction. Quantification was done by using standard curves made from known concentrations of plasmid DNA containing the respective amplicon for each set of primers.

**Table 2:** Ingredient composition and nutrient analysis of the experimental diets

Items	LP	HP
	Ingredients	
	As-is basis (g/100 g)	
Pork greaves	11.0	53.3
Brewers rice	50.9	20.0
Lard	11.0	8.50
Rice meal	15.0	6.00
Beet pulp	3.70	5.00
Dicalcium phosphate	3.00	2.10
Yeast	1.00	1.00
Salmon Oil	1.00	1.00
Animal digest^1^	0.89	0.89
Calcium carbonate	0.40	0.50
Bentonite clay	0.50	0.50
Salt	0.81	0.42
Vitamin mix	0.26	0.23
Mineral mix	0.22	0.22
Chorine chloride	0.14	0.14
Lecithine	0.10	0.10
	Nutrient Analysis	
DM (g/100 g)	92.7	96.2
	g/100 g DM	
Ash	5.35	4.75
CP	17.8	50.0
EE	13.6	12.2
CF	1.05	0.91
NFE^2^	62.3	32.2
Insoluble fibre	2.64	8.28
Soluble fibre	2.15	0.27
TDF	4.79	8.56
ME, kJ/100gDM^3^	1850	1833

LP: low protein diet; HP: high protein diet; CP: crude protein; DM: dry matter; EE: ether extract; CF: crude fibre; ME: metabolizable energy; NFE: nitrogen-free extract; TDF: total dietary fibre

^1^Animal digest: a material which results from chemical and/or enzymatic hydrolysis of clean and undecomposed animal tissue [66]

^2^Calculated %NFE = % DM – (% EE+ % CP + % ash + % CF)

^3^Calculated ME = 16.7×g CP + 37.7×g Fat +16.7×g NFE [27]

### Statistical analyses

Statistical analyses were performed with RStudio (The R Foundation for Statistical Computing, version 3.1.0) using the gamm4 package for R (version 0.2–2).

To test the effect of both dietary protein levels on composition and functionality of microbiota in lean and obese dogs and their effect on host metabolism, a general additive mixed model was used: Y = μ + dog + diet + BC + group + D × BC + ε, where μ is the overall mean, Dog a random effect; diet refers to HP or LP; BC is the body condition of the dogs (lean vs. obese), group is the order by which dogs received the diets (groups 1–4) and refers to the carry-over effect, D × BC is the interaction between diet and body condition and refers to the direction or size of the effect of dietary protein on lean and obese animals; and ε is the error term.

Faecal concentration of valerate and p-cresol were only detected in three and one samples, respectively, and hence these parameters were not taken into consideration when performing the statistical analysis.

For the D × BC, the nparcomp package of R was used (version 2.0) for post-hoc test by creating dummy variables and an own contrast matrix.

Statistical significance was accepted at *P* < 0.05.

## Results

### Food, energy and protein intake

Two obese dogs (one from group 3 and one from group 4) were excluded from the study due to injuries not associated with the present study. Significant D × BC interactions were observed on BW and BCS, and post hoc analysis could only detect the differences between lean and obese dogs (Table 3). No difference was observed on food and energy intake between diets and BC (Table 3). Protein intake was significantly higher in HP compared to LP (*P* < 0.001).

**Table 3:** Body weight, body condition score, food intake, protein intake, energy intake and faecal metabolites of lean (*n* = 6) and obese (*n* = 4) dogs fed low protein diet and high protein diet in a crossover design

Item	LP				HP				*P*		
	Lean		Obese		Lean		Obese		Diet	BC	D × BC
	Mean	SD	Mean	SD	Mean	SD	Mean	SD			
BW (kg)^1^	10.6^a^	1.6	15.0^b^	1.8	10.6^a^	1.6	14.1^b^	1.5	0.869	0.219	0.008
BCS	4.7^a^	0.5	8.5^b^	0.6	4.7^a^	0.5	8.0^b^	1.2	1.000	<0.001	0.045
FI(g/d^.^kg^-1.^BW^0.75^)	37.6	9.4	39.8	6.4	32.3	6.8	27.8	13.3	0.126	0.101	0.211
PI (g/d^.^kg^-1.^BW^0.75^)	6.2	1.6	6.6	1.1	15.5	3.3	14.1	5.4	<0.001	0.176	0.415
EI (kJ/d^.^kg^-1.^BW^0.75^)	626	159	666	108	535	113	461	221	0.118	0.094	0.203
Faecal parameters											
pH	6.8	0.2	6.6	0.3	6.8	0.5	6.6	0.2	0.731	0.061	0.846
Ammonia (μmol/g)^2^	181	45	194	45	330	57	327	107	<0.001	0.066	0.779
Acetate (μmol/g)	87.4	12.1	86.7	12.1	101.4	18.9	86.2	32.3	0.230	0.403	0.423
Propionate (μmol/g)	26.8^a^	8.8	35.5^ab^	21.0	40.0^b^	6.9	30.5^ab^	15.1	0.008	0.027	0.017
Butyrate (μmol/g)	27.9	13.8	23.0	2.1	24.2	2.8	21.0	6.8	0.454	0.824	0.821
Isovalerate (μmol/g)	3.2	0.9	4.8	1.0	8.3	1.8	8.7	3.7	<0.001	0.010	0.443
Isobutyrate (μmol/g)	2.0	0.5	2.8	0.9	4.8	1.4	4.9	1.9	<0.001	0.009	0.435
Total SCFA (μmol/g)^3^	151	31	153	12	179	28	151	57	0.164	0.098	0.373
Phenol (μmol/kg)	199	105	208	73	478	142	563	273	0.004	0.097	0.565
Indole (μmol/kg)	585	227	480	108	1233	110	1193	603	0.002	0.260	0.787

BC: body condition; BCS: body condition score; BW: body weight; D × BC: interaction between diet and body condition; EI: energy intake; FI: food intake, g/d: g per day; PI: protein intake, SCFA: short-chain fatty acids

^1^Values within a row not sharing a common superscript are significantly different

^2^μmol/g and μmol/kg is expressed as the concentration to 1 g and 1 kg faecal samples, respectively

^3^Total SCFA = acetate + propionate + butyrate + isobutyrate + isovalerate + valerate

### Faecal metabolites

Faecal concentrations of ammonia (*P* < 0.001), isovalerate (*P* < 0.001), isobutyrate (*P* < 0.001), phenol (*P* = 0.004) and indole (*P* = 0.002) were higher in dogs on HP compared to LP (Table 3). In addition, faecal isovalerate (*P* = 0.010) and isobutyrate (*P* = 0.009) concentrations were higher in obese than lean dogs. A significant D × BC interaction was observed on faecal propionate (*P* = 0.017), however, a carry-over effect was observed for faecal propionate (*P* = 0.049, data not shown). No differences in faecal concentrations of acetate, butyrate, and total SCFA were observed between diets or BC (Table 3).

### Blood parameters

Serum indoxyl sulphate concentration was higher in dogs fed HP than LP (*P* = 0.027) (Table 4). Within the plasma acylcarnitine profile, only 3-OH isovalerylcarnitine (3OH-C5) was higher (*P* = 0.012) for dogs fed HP compared to LP (Table 4).

**Table 4:** Blood parameters of lean (*n* = 6) and obese (*n* = 4) dogs fed low protein diet (LP) and high protein diet (HP) in a crossover design

Item	LP				HP				*P*		
	Lean		Obese		Lean		Obese		Diet	BC	D × BC
	Mean	SD	Mean	SD	Mean	SD	Mean	SD			
Cobalamin (pmol/L)	416	78	357	112	400	57	406	70	0.635	0.139	0.262
Folate (nmol/L)	26.2	6.3	29.7	5.1	28.2	6.7	20.0	6.7	0.588	0.817	0.061
Indoxyl sulphate (mg/dL)	154	106	118	74	362	351	223	128	0.027	0.841	0.454
SCAC (μmol/L)											
C0	19.2	6.9	19.3	10.8	21.5	8.7	21.6	12.8	0.242	0.998	0.996
C2	3.04	0.94	3.64	1.62	4.18	2.40	4.33	2.95	0.198	0.742	0.742
C3	0.138	0.047	0.165	0.070	0.147	0.038	0.203	0.103	0.690	0.918	0.382
C4	0.080	0.030	0.085	0.052	0.100	0.016	0.085	0.029	0.114	0.531	0.305
C5	0.075	0.023	0.143	0.118	0.100	0.036	0.150	0.114	0.060	0.908	0.381
3OH-C4	0.023	0.005	0.028	0.017	0.035	0.026	0.038	0.029	0.191	0.948	0.903
3OH-C5	0.030	0.006	0.040	0.022	0.047	0.008	0.055	0.021	0.012	0.356	0.859
C3DC	0.023	0.008	0.025	0.013	0.020	0.009	0.023	0.005	0.429	0.761	0.900

BC: body condition; D × BC: interaction between diet and body condition; SCAC: short-chain acylcarnitines; C0: free carnitine; C2: acetylcarnitine; C3: propionylcarnitine; C4: butyryl- and isobutyrylcarnitine; C5: isovalerylcarnitine, 3OH-C4: 3-OH butyrylcarnitine; 3OH-C5: 3-OH isovalerylcarnitine; C3DC: malonylcarnitine

### Microbial ecology and populations

**Fig. 1:**
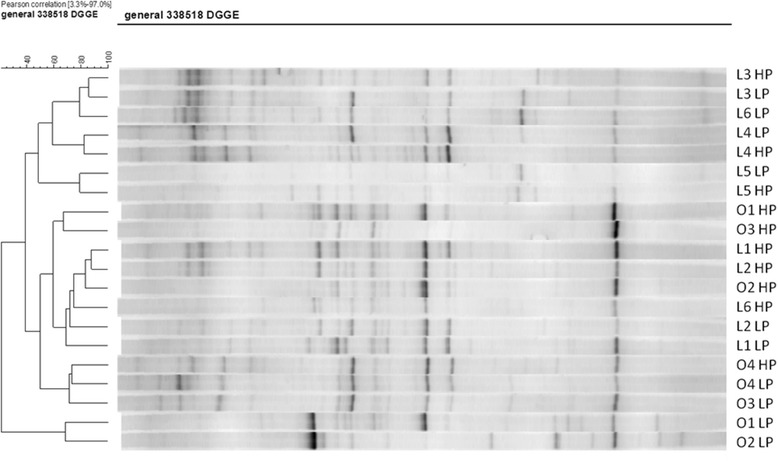
Dice cluster analysis of the DGGE gel profile of lean (L, *n* = 6) and obese (O, *n* = 4) dogs fed low protein diet (LP) and high protein diet (HP) in a crossover design. DGGE: denaturing gradient gel electrophoresis; LP: low protein diet; HP: high protein diet; L: lean dog; O: obese dog (Additional file 1)

**Fig. 2:**
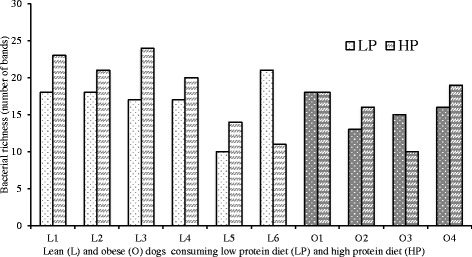
The richness of faecal microbiota in each dog fed LP and HP. LP: low protein diet; HP: high protein diet; L: lean dog; O: obese dog

**Fig. 3:**
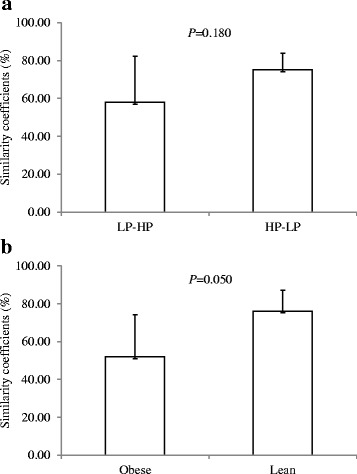
Similarity coefficients of DGGE band patterns from comparison of microbial communities of (a) dogs consuming low protein diet (LP) then high protein diet (HP) vs. dogs consuming HP then LP; (b) obese dogs vs. lean dogs. DGGE: denaturing gradient gel electrophoresis

Neither the type of diets nor the individual played an important role in the clustering of the microbial community (Fig. 1). Although seven out of ten dogs had greater richness when fed HP compared to LP irrespective of BC, no significant difference was observed between diets and between lean and obese dogs. The average value of faecal microbial richness was 17.0 in this study (Fig. 2). The order of the diet (effects of group) did not affect the similarity coefficient of faecal microbiota (Fig. 3a). However, faecal microbiota of obese dogs had a lower similarity value compared to lean dogs (52% vs. 76%) when the two diets were switched (*P* = 0.050) (Fig. 3b).

Higher faecal abundances of clostridial cluster IV (*P* = 0.025), XIVa (*P* = 0.001) and of the BK gene (*P* = 0.019) were observed when dogs were fed HP compared to LP (Table 5). In addition, a significant D × BC interaction was observed for Firmicutes (*P* = 0.007), *Lactobacillus* (*P* = 0.017) and clostridial cluster I (*P* = 0.022). Compared to LP, HP increased the abundance of Firmicutes in lean dogs (*P* < 0.05), while no changes were observed in obese dogs. Clostridial cluster I was more abundant in lean dogs when fed HP than LP (*P* < 0.05), whereas post-hoc test did not reveal any differences between the four groups for *Lactobacillus*. No effects of diet or BC were found for faecal concentrations of total bacteria, Bacteroidetes, Enterobacteriaceae and gene express of BCoAT.

**Table 5:** The abundance of different bacterial groups and functional genes in lean (*n* = 6) and obese (*n* = 4) dogs fed low protein diet (LP) and high protein diet (HP) in a crossover design^1,2^

Item	LP				HP				*P*		
	Lean		Obese		Lean		Obese		Diet	BC	D × BC
	Mean	SD	Mean	SD	Mean	SD	Mean	SD			
Total bacteria	10.76	0.15	10.64	0.35	11.01	0.08	10.61	0.23	0.126	0.557	0.285
Firmicutes	9.49^a^	0.26	9.60^ab^	0.37	9.88^c^	0.08	9.61^b^	0.17	0.006	0.976	0.007
Bacteroidetes	10.66	0.41	9.54	1.50	11.07	0.43	10.22	0.43	0.287	0.795	0.648
Enterobacteriaceae	7.58	0.95	8.23	0.36	7.30	1.03	8.36	0.74	0.488	0.597	0.526
*Lactobacillus*	7.48	1.00	8.00	1.43	7.84	1.31	6.92	1.22	0.306	0.402	0.017
Clostridial cluster I	8.76^a^	0.13	9.33^ab^	0.72	9.29^b^	0.29	9.01^ab^	0.17	0.025	0.954	0.022
Clostridial cluster IV	7.65	0.28	7.61	0.39	8.17	0.19	7.86	0.44	0.025	0.354	0.432
Clostridial cluster XIVa	8.80	0.20	9.20	0.30	9.60	0.30	9.40	0.20	0.001	0.160	0.095
Butyryl-CoA acetate-CoA transferase	6.82	0.72	6.59	0.73	7.16	0.50	6.74	0.20	0.139	0.988	0.426
Butyrate kinase	5.79	0.24	6.81	0.25	6.34	0.77	6.67	0.24	0.019	0.877	0.334

BC: body condition; D × BC: interaction between diet and body condition

^1^The abundance of bacterial groups was expressed as log_10_ 16S rRNA gene copies / g of fresh faeces and of functional gene was expressed as log_10_ gene copies of total DNA / g of fresh faeces

^2^Values within a row not sharing a common superscript are significantly different

## Discussion

Understanding how dietary components alter the composition and activity of gut microbiota direct nutritional interventions and disease prevention strategies. The present study has demonstrated an impact of dietary protein content on both composition and activity of faecal microbiota in dogs. In particular, the major butyrate producing bacterial groups, *Clostridial* cluster IV and XIVa, and BK, the terminal gene of butyrate production were increased in dogs fed HP. Interestingly the present study has also shown a different response to dietary protein content in lean and obese dogs, which to the author’s knowledge has not yet been reported. In previous studies, human obesity has been associated with a number of changes in faecal microbial groups, such as consistently reported changes in two predominant phyla Firmicutes and Bacteroidetes, which have been suggested to be important for energy harvest [5, 6]; and an increase in the family Enterobacteriaceae, an important producer of inflammatory lipopolysaccaride [37]. In addition, decreases in butyrate producing bacteria have been reported in obese humans with reduced carbohydrate intake [24]. Therefore, these bacterial groups were selected and measured in the present study. Surprisingly, in contrast to humans, increases in the abundance of major butyrate-producing bacterial groups, clostridial clusters IV and XIVa were observed in this study. However, clostridial clusters IV and XIVa still harbour non-butyrate-producing bacteria and some butyrate-producing strains within these clusters (eg. *Coprococcus catus* and *Roseburia inulinivorans*) can switch from butyrate to propionate production [38, 39]. When measuring the terminal genes of the two butyrate production pathways, the BK gene was increased whereas BCoAT remained unchanged in dogs fed HP. This is in contrast to humans where the BCoAT pathway has been found to be the dominant pathway for butyrate formation [23]. Possible reasons for dogs utilizing a different butyrate synthesis pathway compared to humans are explained below.

The accumulation of butyrate-producing bacteria is often associated with intake of fibre. In this study, TDF in HP was two-fold higher compared to LP. Generally, TDF is comprised of plant fibre which in this study was provided by brewer’s rice, rice meal, and beet pulp (Table 1), with TDF ranging from 1.6–16.4% [40, 41], 2.4–4.6% [27], and 60–80% [42], respectively. However, TDF does not clearly differ from HP to LP in terms of plant fibre content. Therefore, the higher TDF content in HP vs. LP could be of animal origin [31, 43], where the content of pork greaves was clearly higher in HP vs LP. TDF in the pork greaves was thus analyzed and observed to be 7.0% on DM basis.

Although TDF was higher, soluble fibre was much lower in HP compared to LP. In contrast to insoluble fiber which cannot be fermented, soluble fibre is readily fermentable [44]. Thus, it is unlikely that the dietary fibre contributed to the increase in butyrate producing bacteria. Importantly, butyrate can be formed by fermentation of amino acids, such as glutamate and leucine [45]. Thus, in the present study, amino acids might be the important substrates for the growth of butyrate producing bacteria. This is supported by increased BK gene expression because BK pathway might be associated more with protein-rich environment, whereas BCoAT pathway is depending on a consistent supply of acetate that is derived from carbohydrates [46]. Recent studies have found that the BK gene was more abundant in carnivorous animals and BCoAT gene was enriched in omnivores and herbivores [46].

As dogs may use a different pathway for butyrate production, it raises the question whether dogs possess the same predominant butyrate producers as humans (clostridial clusters IV and XIVa). The butyrate kinase gene pathway is linked to *C. perfringens* dominated in many Carnivores (e.g. ferret, tiger, African lion) and non-carnivorous Carnivora (e.g. red panda and giant panda) [46]. In this study *C. perfringens* was not measured, but it is the major component of clostridial cluster I. The clostridial cluster I was increased when dogs were fed HP, however, it still needs to be confirmed if increased clostridial cluster I abundance is due to increases in *C. perfringens* numbers. Nevertheless, other canine studies have also shown that high protein diets promoted the growth of *C. perfringens* [12]. Thus, closteridial cluster I might also be important for butyrate production in dogs. Further studies are warranted to investigate the diversity, metabolism and microbial ecology of butyrate-producing bacteria from the dog gut.

In contrast to our results, another study reported high dietary protein decreased the abundance of clostridial cluster XIVa in dogs [47]. This contradiction might be due to the different quality/components in the greaves meal (e.g. bone and cartilage), and/or different level of greaves meal included in the diet, 80.0% in that study vs. 53.4% in our study, in particular, diarrhea has been observed in that study, which possibly indicates gut dysbiosis in those dogs fed that diet. Furthermore, faecal butyrate concentrations and plasma C4 concentrations did not differ between the two diets, this could be due to the fact that faecal concentrations may not necessarily reflect SCFA production in the proximal colon because more than 95% of SCFA are absorbed rapidly [39]. Nevertheless, a recent study observed an increased butyrate concentration in a high minced beef diet compared to commercial dry food, whereas protein content was 46.2% versus 27.1% on dry matter basis [48]. Moreover, the technique used to measure acylcarnitines could not separate butyrylcarnitine and isobutyrylcarnitine. Therefore, the accurate estimation of in vivo butyrate production is hardly feasible in dogs.

Although several studies have shown that high protein diets can alter faecal microbiota in obese human subjects [24], whether the diet independent of the obese phenotype is responsible for the changes remains largely unknown. One study reported an increase in Enterobacteriales in the faecal microbiota of obese but not of lean rats [49]. In the present study, the similarity coefficient of faecal microbiota between the two diets was lower in obese dogs than in lean dogs (Fig. 2b), suggesting faecal microbiota in lean dogs might be more resilient to dietary protein changes than in obese dogs. This is in accordance with another study that found dietary protein and carbohydrate ratios have more significant impacts on gut microbial compositions in obese dogs than in lean dogs [50]. In addition, within the quantified bacterial groups, a high protein diet promoted bacterial growth, i.e. Firmicutes, *Lactobacillus* and clostridial cluster I in lean dogs whereas no changes were observed obese dogs (Table 5). Therefore, dietary modulation on faecal microbiota was also affected by body condition in dogs.

As expected, colonic protein fermentation was increased in dogs fed HP as indicated by higher faecal concentrations of putrefactive compounds (ammonia, indole and phenol) and branched-chain fatty acids (isovalerate and isobutyrate) compared to dogs consuming LP. These results are in agreement with previous studies in dogs fed high protein diets [11, 51]. In addition, higher serum concentrations of indoxyl sulphate and 3-OH isovalerylcarnitine in dogs fed HP were observed, supporting colonic production and absorption of protein fermentation metabolites [45]. To date, no toxicity or tolerance tests have been performed on the effects of protein fermentation metabolites on canine gut health, and the only studies available concerning the link between protein fermentation and canine gut health have evaluated the effects of prebiotics, probiotics and synbiotics [52, 53]. Studies have found *Novosphingobium sp.* and *Haliangium ochraceum*, which are capable of breaking down aromatic putrefactive substances were increased in cats fed a high protein diet [54]. Therefore, whether the increased protein fermentation metabolites are harmful for dogs needs to be further investigated, especially the beneficial effects of indole that have been proposed by in vitro studies, such as the increase in the expression of anti-inflammatory genes and strengthening of epithelial cell barrier properties [55].

## Conclusions

In our study, high protein diet promoted the growth of butyrate-producing bacteria which includes clostridial clusters I, IV and XIVa in dogs independent of body condition, and importantly this butyrate synthesis is suggested to relate to the BK pathway as compared to BCoAT pathway in humans. Thus, the different metabolic pathway used for butyrate production provides valuable information for modulation of gut microbiota and their fermentation metabolites in dogs. In addition, HP induced several bacterial changes (including Firmicutes, *Lactobacillus* and clostridial cluster I) that are body condition dependent. Further, faecal microbiota in obese dogs seemed to be less stable compared to that of lean dogs. Together, the results of the present study suggest that diet composition per se had an important effect on the faecal microbiota, however, body condition also affected microbiota composition in dogs. This should be taken into consideration in future nutritional interventions and disease prevention.

## Additional file

Additional file 1: The DGGE gel of the present study. Description of data: the original DGGE gel of the present study that includes the markers, blanks, and 20 faecal samples. (PNG 1049 kb)

## Abbreviations

3OH-C4: 3-OH butyrylcarnitine
3OH-C5: 3-OH isovalerylcarnitine
BC: Body condition
BCoAT: Butyryl CoA: acetate CoA transferase
BCS: Body condition score
BK: Butyrate kinase
BW: Body weight
C0: Free carnitine
C2: Acetylcarnitine
C3: Propionylcarnitine
C3DC: Malonylcarnitine
C4: Butyryl- and isobutyrylcarnitine
C5: Isovalerylcarnitine
CoA: Co-enzyme A
CP: Crude protein
D × BC: Interaction between diet and body condition
DGGE: Denaturing gradient gel-electrophoresis
DM: Dry matter
EE: Ether extract
EI: Energy intake
FI: Food intake
HP: High protein diet
LP: Low protein diet
NFE: Nitrogen free extract
PI: Protein intake
SCFA: Short-chain fatty acids
TDF: Total dietary fibre
