# Supplementary figures and images for: The response of canine faecal microbiota to increased dietary protein is influenced by body condition
Source: BMC Vet Res. 2017 Dec 4;13:374. doi: 10.1186/s12917-017-1276-0 (PMC5716228; doi:10.1186/s12917-017-1276-0)

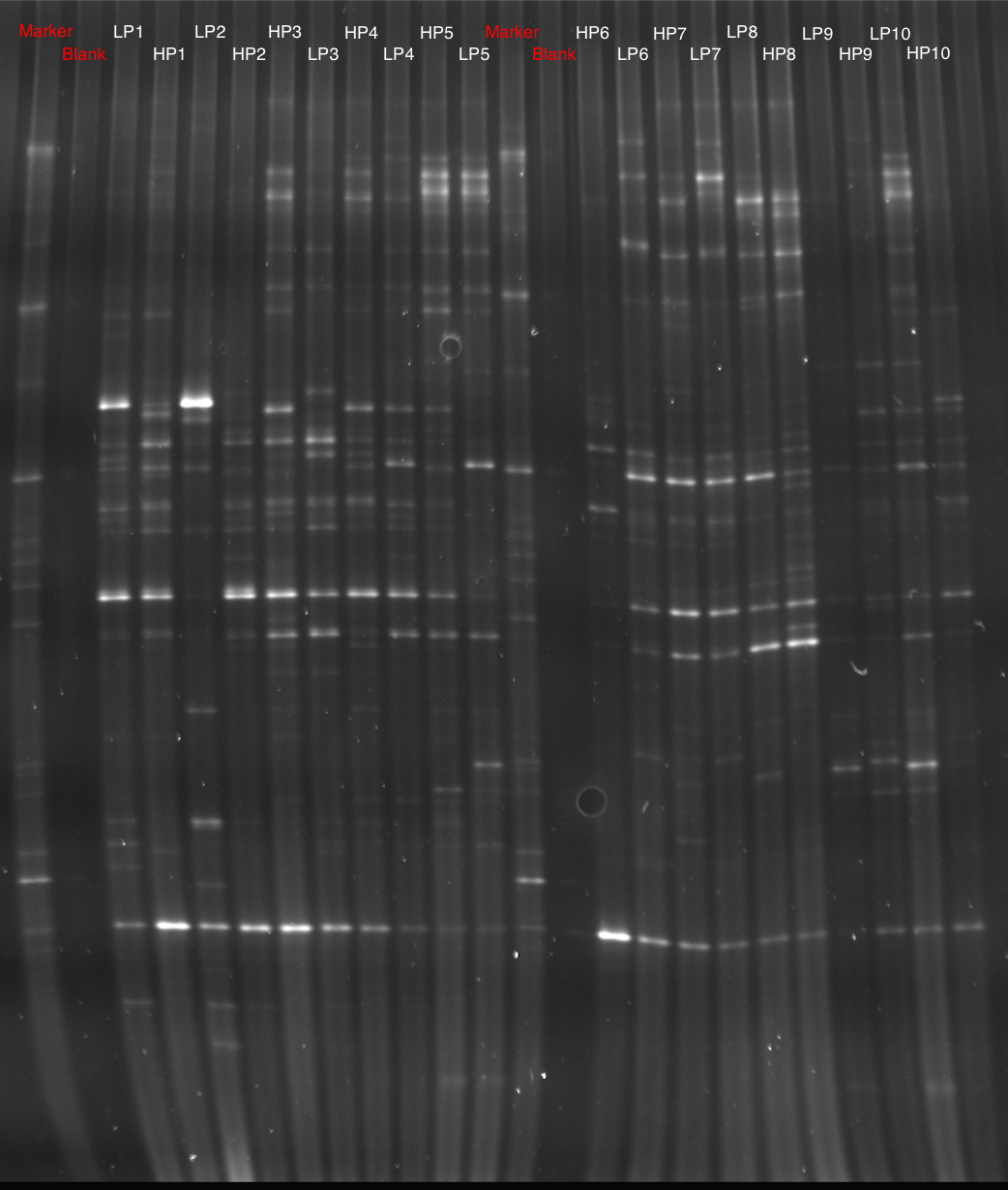

Supplement: Additional file 1: — The DGGE gel of the present study. Description of data: the original DGGE gel of the present study that includes the markers, blanks, and 20 faecal samples. (PNG 1049 kb) [file 12917_2017_1276_MOESM1_ESM.png]
